# The Influence of Gene Aberrations on Survival in Resected *IDH* Wildtype Glioblastoma Patients: A Single-Institution Study

**DOI:** 10.3390/curroncol28020122

**Published:** 2021-03-21

**Authors:** Ondrej Kalita, Zuzana Sporikova, Marian Hajduch, Magdalena Megova Houdova, Rastislav Slavkovsky, Lumir Hrabalek, Matej Halaj, Yvona Klementova, Martin Dolezel, Jiri Drabek, Lucie Tuckova, Jiri Ehrmann, Jana Vrbkova, Radek Trojanec, Miroslav Vaverka

**Affiliations:** 1 Department of Neurosurgery, University Hospital Olomouc, I.P. Pavlova 6, 779 00 Olomouc, Czech Republic; lumir.hrabalek@fnol.cz (L.H.); matej.halaj@fnol.cz (M.H.); miroslav.vaverka@fnol.cz (M.V.); 2 Institute of Molecular and Translational Medicine, Faculty of Medicine and Dentistry, Palacky University in Olomouc, Hnevotinska 5, 779 00 Olomouc, Czech Republic; sporikovaz@gmail.com (Z.S.); marian.hajduch@fnol.cz (M.H.); magdalena.houdova-megova@lf1.cuni.cz (M.M.H.); rastislav.slavskovsky@upol.cz (R.S.); jiri.drabek@upol.cz (J.D.); jana.vrbkova@upol.cz (J.V.); radek.trojanec@fnol.cz (R.T.); 3 Department of Oncology, University Hospital Olomouc, I.P. Pavlova 6, 779 00 Olomouc, Czech Republic; yvona.klementova@fnol.cz (Y.K.); martin.dolezel@fnol.cz (M.D.); 4 Department of Pathology and Laboratory of Molecular Pathology, University Hospital Olomouc, Hnevotinska 3, 779 00 Olomouc, Czech Republic; lucie.tuckova@fnol.cz (L.T.); jiri.ehrmann2@fnol.cz (J.E.J.)

**Keywords:** glioblastoma, biomarkers, multimodal therapy

## Abstract

This prospective population-based study on a group of 132 resected IDH-wildtype (IDH-wt) glioblastoma (GBM) patients assesses the prognostic and predictive value of selected genetic biomarkers and clinical factors for GBM as well as the dependence of these values on the applied therapeutic modalities. The patients were treated in our hospital between June 2006 and June 2015. Clinical data and tumor samples were analyzed to determine the frequencies of TP53, MDM2, EGFR, RB1, BCR, and CCND1 gene aberrations and the duplication/deletion statuses of the 9p21.3, 1p36.3, 19q13.32, and 10p11.1 chromosome regions. Cut-off values distinguishing low (LCN) and high (HCN) copy number status for each marker were defined. Additionally, MGMT promoter methylation and IDH1/2 mutation status were investigated retrospectively. Young age, female gender, Karnofsky scores (KS) above 80, chemoradiotherapy, TP53 HCN, and CCND1 HCN were identified as positive prognostic factors, and smoking was identified as a negative prognostic factor. Cox proportional regression models of the chemoradiotherapy patient group revealed TP53 HCN and CCND1 HCN to be positive prognostic factors for both progression-free survival and overall survival. These results confirmed the influence of key clinical factors (age, KS, adjuvant oncotherapy, and smoking) on survival in GBM IDH-wt patients and demonstrated the prognostic and/or predictive importance of CCND1, MDM2, and 22q12.2 aberrations.

## 1. Introduction

Glioblastoma (GBM) is a highly invasive tumor type that is not amenable to complete resection [1,2,3]. Consequently, patients with GBM have a poor prognosis, with a median survival of only about 12 months. Advanced age, male gender, Caucasian race, exposure to ionizing radiation, and smoking are considered risk factors for glioma development [4,5]. Conversely, clinical factors including young age, good performance status, gross total resection, and adjuvant treatment have been linked to superior prognosis among unselected GBM patients [6,7,8,9]. 

Glioblastomas can be classified based on their isocitrate dehydrogenase (IDH) status. GBM with wt IDH is characterized by tumor protein p53 (TP53) mutations, phosphatase tensin homolog (PTEN) mutations and/or complete loss of chromosome 10, homozygous deletion of cyclin-dependent kinase inhibitor 2A/2B (CDKN2A/CDKN2B), mutation or overexpression of epidermal growth factor receptor (EGFR), and/or gain of the 7p chromosome arm, platelet-derived growth factor receptor 1 (PDFGRA1) mutations, neurofibromin 1 (NF1) mutations, E3 ubiquitin-protein ligase (MDM2) amplification, and methylguanine-DNA methyltransferase (MGMT) promoter methylation [10]. 

The objective of this population-based, single-center study was to determine the influence of selected genetic aberrations and clinical factors on survival in defined resected GBM IDH wt patients receiving different post-resection treatments. The WHO 2007 classifications initially used in the clinical setting were converted into WHO 2016 IDH status classifications in accordance with current recommendations [11,12].

## 2. Materials and Methods

### 2.1. Patients

**Figure 1:**
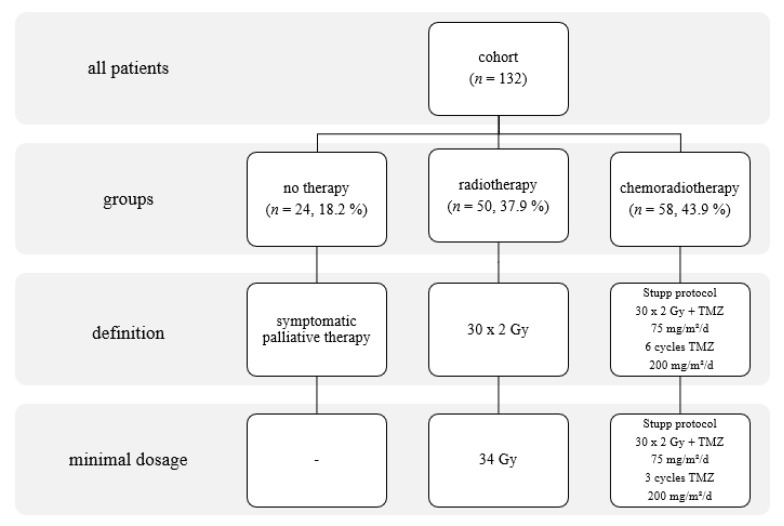
Stratification of the cohort based on treatment modality, with details of the applied treatments.

Data on all glioma patients treated at the University Hospital in Olomouc, Czech Republic, have been collected prospectively and systematically since 2006. This work focuses on adult supratentorial IDH wt GBM patients who underwent resection and oncotherapy between June 2006 and June 2015. Information on the patients’ clinical condition (KS, PS WHO, and smoking status) was collected along with imaging and histological data on the tumors and details of their cytogenic alterations. All patients underwent early post-surgical MRI (within 72 h) to determine resection radicality. Nineteen patients received second-look surgery in the week after the initial procedure. Removal of at least 80% of the original tumor volume was considered to be the lowest acceptable resection radicality for inclusion in this study [13,14]. Based on semi-automatic MRI analyses, the extent of resection of the contrast-enhanced tumor was 84–100%, with a median of 85.6%. The median of the postoperative tumor volume was 2.9 cc. We have strived to perform standard aggressive oncotherapy in all patients. But in real clinical practice, only some patients are eligible to undergo Stupp protocol. Many patients have progressed before and at the initial period of oncotherapy. We separated two groups of patients named No Therapy and Chemoradiotherapy. Finally, we singled out patients that were eligible to go through, only semi-palliative treatment strategy characterized by sole radiotherapy ranged from 34 to 60 Gy [15]. The stratification of the cohort based on the applied oncologic treatment strategy is shown in Figure 1. Following resection, patients received periodic checkups with MRI every 3 months until death. 

Tumor tissue samples were collected in both formalin-fixed paraffin-embedded (FFPE) and fresh-frozen form. Clinical and MRI follow-up information was gathered at regular three-monthly intervals. Smoking status was obtained from the clinic’s preoperative questionnaire. Patients were classified as non-smokers (83/132 = 62.9%) if they had never smoked or had quit smoking at least five years before diagnosis; the remaining patients (49/132 = 37.1%) were classified as smokers. All of the tumors were classified according to the latest WHO classification of CNS tumors by two local neuropathologists. Immunohistochemical analysis of IDH1 R132H and Ki67 status was included in the standard classification procedure. All patients signed informed consent forms.

### 2.2. Immunohistochemical Determination of IDH1 R132H Status

The study cohort consisted of GBM patients with IDH wildtype status according to the WHO 2016 diagnostic criteria. The 1–2 μm thick tissue sections were pre-treated using the PT Link system (Agilent, Santa Clara, CA, USA) at 97 °C, pH 9 for 20 min to ensure epitope retrieval. Hydrogen peroxide was used to block endogenous peroxidase activity. The sections were subsequently treated with the primary antibody, Anti-IDH1R132H clone H09 (Dianova, Hamburg, Germany), at a dilution of 1:100 for 20 min at room temperature. EnVision Flex+, Mouse, High pH (Agilent DAKO) was used to amplify the primary antibody signal. The sections were then treated for 20 min with the secondary antibody, EnVision Flex/HRP (Agilent DAKO), after which the reaction was visualized using the DAB+ Substrate Chromogen System (Agilent DAKO).

### 2.3. Immunohistochemical Determination of Ki67 Status

The 1–2 μm thick tissue sections were microwaved in citrate buffer (pH 6) for 10 min at 120 °C to ensure antigen retrieval. Hydrogen peroxide was used to block endogenous peroxidase activity (Mouse/Rabbit ImmunoDetector DAB HRP Brown System, Bio SB, Santa Clara, CA, USA). The sections were subsequently treated for 30 min with the primary antibody, Ki67 antigen, MIB-1 (Agilent DAKO), at a dilution of 1:200. After treatment with the secondary antibody (Mouse/Rabbit ImmunoDetector DAB HRP Brown System, Bio SB) for 30 min, the reaction was visualized using DAB+ Substrate Chromogen.

### 2.4. IDH1 R132 and IDH2 R172 Genotyping

DNA was isolated using a Roche Cobas^®^ kit and a Cobas^®^ DNA sample preparation kit following the manufacturer’s instructions. qPCR amplification was performed using 1 μL or 5 μL total DNA (depending on whether the DNA concentration was >30 ng/μL or >10 ng/μL) with Thermo-Start Taq DNA Polymerase 1U (ThermoFisher Scientific, Weltham, MA, USA), 10X PCR Thermo-Start Buffer (ThermoFisher Scientific), MgCl2 (ThermoFisher Scientific), dNTPs (Meridian Bioscience, Cincinnati, OH, USA), and EvaGreen 20x (Biotium, Fremont, CA, USA). The conditions used for multiplex PCR amplification (IDH1 and IDH2 reactions) were as follows: 95 °C for 2 min 15 s, then 40 cycles of 15 s at 95 °C, 30 s at 62 °C, and 30 s at 72 °C (FAM channel fluorescence scanning), followed by a final incubation for 5 min at 72 °C and melting from 60 °C to 95 °C while scanning fluorescence in the FAM channel at 0.5 °C. After amplification, the QIAquick PCR Purification Kit (Qiagen, Hiden, Germany) was used to purify amplicons. The product concentration was determined using the Qubit 2.0 HS DNA kit (Invitrogen, Carlsbad, CA, USA). The sequencing library was diluted and denatured with 0.1 M NaOH (DNA concentration: 10 μM). The library contained approximately equal quantities of each sample. Sequencing was performed with the Illumina MiSeq platform using the MiSeq V2 Nano 300 bp or V3 150 bp sequencing kit and 2 × 75 bp sequencing reads. Sequencing results were analyzed using the Somatic Variant Caller function of the MiSeq Reporter software package. Output vcf files were processed using Excel (Microsoft, Redmont, WA, USA). Mutations in the IDH1 and IDH2 genes were identified by searching for mutations in codons 132 and 172 with frequencies >5% and coverage values >1000.

### 2.5. FISH Analysis

FISH analysis was performed on FFPE tissues according to the manufacturer’s instructions with LSI 1p36.3, LSI 1q25.2, LSI 9p21.3, LSI 19q13, LSI EGFR, LSI CEP7, LSI MDM2, LSI 10p11.1, LSI BCR, LSI 22q12.2, and LSI CCND1 probes from IntellMed, Ltd. (Olomouc, Czech Republic) as well as LSI TP53, LSI RB1, and LSI 13q12.11 probes from Vysis (Lake County, IL, USA). Signals were detected and quantified using fluorescence microscopy. At least 100 non-overlapping nuclei were inspected in each sample. 

### 2.6. Setting Copy Number Cutoff Values

Current therapeutic protocols for gliomas lack standard cutoff values for molecular aberrations. Therefore, a specific cut-off value for each molecular marker was defined by selecting the best cut-off value found in our dataset using the maxstat function (maxstat R package, ver. 0.7–25) and the surv_cutpoint function (survminer R package, ver. 0.4.3) with the default value of 0.1 for the minprop parameter (representing the minimal proportion of observations per group) and data on progress-free survival (time and event). Two patient groups were defined for each marker: a low copy number (LCN) group comprising patients with copy numbers equal to or below the estimated cutoff value, and a high copy number (HCN) group comprising patients with copy numbers exceeding the cutoff value.

### 2.7. MGMT Methylation Status

Bisulfite conversion of template DNA was performed using the EZ DNA methylation Gold Kit according to the manufacturer’s instructions (Zymo Research, Irvine, CA, USA) immediately after extracting DNA from glioma tissue samples using the DNA Sample Preparation Kit (Roche, Pleasanton, CA, USA). MGMT methylation was then detected using MethyLight real-time methylation-specific PCR. To verify DNA integrity and the quality of the bisulfite conversion and the PCR reaction, the methylation of CDH1 (E-cadherin) and Alu-M5 was analyzed in parallel with that of MGMT, and a commercial methylated and bisulfite-converted DNA standard (Zymo Research) was analyzed as a control alongside all DNA extracts from tissue samples. 

Each 10 µl PCR reaction mixture for detection of MGMT, E-cadherin, and Alu-M5 promoter methylation contained 1x PCR buffer (Qiagen, Hiden, Germany), 1 mM MgCl2, 0.2 mM dNTPs, 0.5 U HotStarTaq (Qiagen, Hiden, Germany), and the relevant primers and probe (Table 1). The PCR program involved denaturation at 95 °C for 15 min then 40 cycles of 95 °C for 30 s, 65 °C for 50 s, and 72 °C for 60 s.

### 2.8. Statistical Analysis 

**Figure 2:**
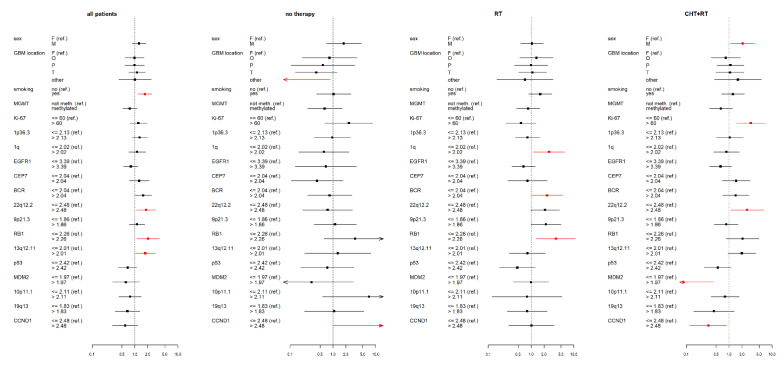
Results of the multivariate Cox proportional hazard models of overall survival (OS) for studied variables (selected clinical factors and molecular markers) in therapy subgroups, expressed using a hazard ratio (a square point—the point estimation, a line segment corresponding to the 95% confidence interval; in case of wider interval than presented scale the arrow is used) for each level of presented factors. The reference category of each factor is labeled with “(ref.)”. Each factor was analyzed in a separate model with adjusting variables—categorized age and Karnofsky score (HRs are not presented). Significant results (*p* < 0.05) are shown in red; the dotted line indicates a hazard ratio of 1. RT—radiotherapy; CHT+RT—chemoradiotherapy; F—female; M—male; F—frontal; O—occipital; P—parietal; T—temporal; BCR—breakpoint cluster region; EGFR1—epidermal growth factor receptor 1; RB1—retinoblastoma gene 1; TP53 - tumor protein P53; MDM2—mouse double minute 2 homolog; CCND1—cyclin D1; MGMT - O6-methylguanine-DNA methyltransferase.

**Figure 3:**
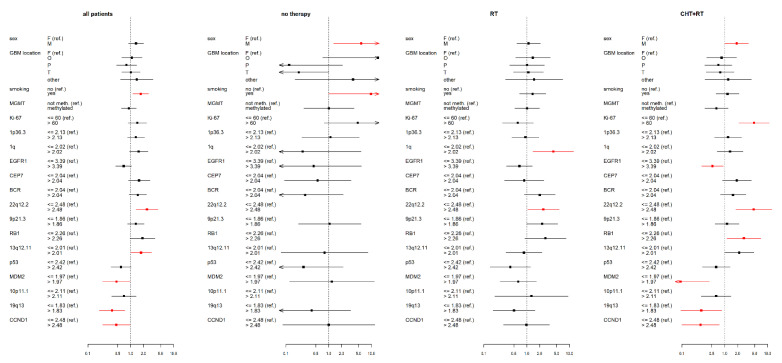
Results of the multivariate Cox proportional hazard models of progression-free survival (PFS) for studied variables (selected clinical factors and molecular markers) in therapy subgroups, expressed using a hazard ratio (a square point—the point estimation, a line segment corresponding to the 95% confidence interval; in case of wider interval than presented scale the arrow is used) for each level of presented factors. The reference category of each factor is labeled with “(ref.)”. Each factor was analyzed in a separate model with adjusting variables—categorized age and Karnofsky score (HRs are not presented). Significant results (*p* < 0.05) are shown in red; the dotted line indicates a hazard ratio of 1. RT—radiotherapy; CHT+RT—chemoradiotherapy; F—female; M—male; F—frontal; O—occipital; P—parietal; T—temporal; BCR—breakpoint cluster region; EGFR1—epidermal growth factor receptor 1; RB1—retinoblastoma gene 1; TP53—tumor protein P53; MDM2—mouse double minute 2 homolog; CCND1—cyclin D1; MGMT—O6-methylguanine-DNA methyltransferase.

All statistical analyses were performed using R Statistical Software, version 4.0.3 (www.r-project.org), accessed date 9 November 2020. Pearson’s chi-square and Fisher’s exact tests were used for testing associations between a type of therapy and molecular markers value levels. Overall survival (OS) and progress-free survival were estimated using the Kaplan-Meier method (presented as medians in tables of results). The OS range extended from the day of the first surgery until death or last follow-up. The PFS range extended from the day of the first surgery until MRI tumor progression. The influence of each molecular marker on OS, resp. PFS was investigated using Cox proportional hazard models, both with one factor (a univariate model—Table S1, Figure 2 and Figure 3) as well as adjusted for the major clinical prognostic factors (a multivariate model—Table S2), i.e., a categorized age at diagnosis (≤55 vs. >55 years) and a categorized Karnofsky score (KS; <80 vs. ≥80), in therapy-subgroups of patients. The influence of each factor on OS, resp. PFS across groups of all patients was investigated by the Cox proportional hazard model (one for each marker) stratified by therapy and adjusted for categorized age and categorized Karnofsky score (Table S3).

## 3. Results

### 3.1. Baseline Patient Characteristics

The studied GBM IDH wt cohort comprised 132 patients who had undergone resection. Each patient was followed up at 3-monthly intervals until their death; the median follow-up period was 7.6 months (1.0–113.7 months). Table 2 summarizes the clinical characteristics of the patients (gender, age, KS, location, smoking, and adjuvant therapy) and their molecular cytogenetic characteristics, namely their high or low copy number (HCN or LCN) status with respect to the studied genetic aberrations and their MGMT promoter methylation status. 

### 3.2. IDH wt GBM Patients Receiving Chemoradiotherapy

Multivariate analysis revealed a significant link between gender and survival among patients receiving chemoradiotherapy. Male gender had negative impacts on PFS (HR = 1.9; *p* = 0.046) and OS (HR = 2.1; *p* = 0.03).

High Ki67 expression was associated with shorter OS (median = 7.9 months vs. 15 months for lower expression of Ki67; HR = 2.9, *p* = 0.005) and PFS (median = 4.4 months vs. 9.6 months for lower expression of Ki67; HR = 4.5, *p* = <0.001) in the chemoradiotherapy group. The same effect was observed in the case of the Cox model adjusted for categorized age and Karnofsky score (HR(OS) = 3.2, *p*-value = 0.003; HR(PFS) = 4.8, *p*-value < 0.001).

Furthermore, 22q12.2 HCN patients had shorter PFS than their LCN counterparts (median = 6.1 months vs. 9.7 months; *p* = 0.006), which was confirmed by multivariate analysis (HR = 4.8; *p* = 0.002). The multivariate analysis adjusted for age and Karnofsky score also indicated that 22q12.2 HCN is linked with a poor OS (HR = 2.6; *p* = 0.033).

CCND1 HCN predicted longer PFS in the chemoradiotherapy group (median = 13.3 months vs. 6.9 months in CCND1 LCN patients; *p* = 0.015) and was associated with reduced relative risk (HR = 0.3; *p* = 0.011). CCND1 HCN was also associated with longer OS (median = 18.9 months vs. 13.6 months for CCND1 LCN; *p* = 0.029), which was confirmed by a low HR (HR = 0.3, *p* = 0.026)

19q13 HCN status was associated with longer PFS (median = 8.9 months vs. 5.5 months for 19q13 LCN; *p* = 0.037) and reduced relative risk (HR = 0.3; *p* = 0.025). 

MDM2 HCN was significantly associated with longer PFS (median = 8.8 months vs. 3.9 months in MDM2 LCN patients; *p* < 0.001) and OS (median = 14.8 months vs. 5.2 in MDM2 LCN patients; *p* < 0.001) in the chemoradiotherapy group. This finding was supported by the multivariate analysis: the HR of MDM2 was below unity for both PFS (HR = 0.1; *p* = 0.002) and OS (HR = 0.1; *p* = 0.003).

Finally, p53 HCN was significantly associated with longer OS (median = 18.9 months vs. 13.6 in p53 LCN; *p* = 0.032) in the chemoradiotherapy group, although this finding was not confirmed by multivariate analysis. However, multivariate analysis did indicate a possible link between RB1 HCN and PFS (HR = 2.8; *p* = 0.026) in this patient group.

### 3.3. IDH wt GBM Patients Receiving Radiotherapy

1q HCN was significantly associated with decreased OS (median = 3.9 months vs. 8.9 months in 1q LCN patients; *p* = 0.022) and PFS (median = 2.7 months vs. 4.6 months in 1q LCN patients; *p* = 0.019) as well as a high relative risk (HR) with respect to both OS (HR = 2.7; *p* = 0.022) and PFS (HR = 4.2; *p* = 0.01) in the radiotherapy patient group based on multivariate models. 

22q12.2 HCN was found to be a negative prognostic factor of PFS (median PFS was 2.3 months vs. 3.6 in 22q12.2 LCN patients; *p* = 0.042). The multivariate model for this marker confirmed an increased relative risk (HR = 2.4; *p* = 0.038). 

BCR HCN was associated with shorter OS (median = 5.3 months vs. 8.5 months in BCR LCN patients; *p* = 0.039), which was confirmed by the multivariate analysis (HR = 2.4; *p* = 0.042). 

RB1 HCN was also associated with shorter OS (median = 3.6 months vs. 6.9 in RB1 LCN patients; *p* = 0.012), and this association was confirmed by its relative risk estimate (HR = 3.8; *p* = 0.013).

### 3.4. IDH wt GBM Patients with No Therapy

Because the group with untreated wt-GBM comprised only 24 patients, some parameters could not be calculated. Nevertheless, male gender was associated with reduced PFS (median = 0.6 vs. 1.7 months in females; *p* = 0.015) in this group, which was confirmed by a higher relative risk estimate (HR = 5.7; *p* = 0.02). 

A history of smoking was also associated with shorter PFS among patients not receiving therapy (median = 0.7 vs. 1.8 months for non-smokers; *p* = 0.036), but the multivariate analysis did not confirm this finding.

CCND1 HCN was linked to shorter OS (median = 1.3 months vs. 3.1 months in CCND1 LCN patients; *p* = 0.043) in the untreated group and had a HR considerably greater than unity (HR = 12.8; *p* = 0.049). However, the reliability of these values is limited by the low number of cases.

### 3.5. Statistical Data for the Complete IDH wt GBM Cohort

To reveal the influence of each factor on OS, resp. PFS across all therapy-subgroups Cox models stratified by type of applied therapy and adjusted for categorized age and Karnofsky score were fitted. Results showed a higher relative risk estimate for the male gender (HR = 1.6; *p* = 0.029). RB1 HCN was found to be a negative marker for both OS (HR = 2.5; *p* = 0.003) and PFS (HR = 2.6; *p* = 0.006). CCND1 HCN was linked to decreased estimated HR (HR = 0.4; *p* = 0.034) and also for 19q13 HCN, the same impact was observed (HR = 0.4; *p* = 0.012). EGFR1 HCN was also associated with reduced OS (HR = 0.6; *p* = 0.024) along with PFS (HR = 0.6; *p* = 0.007). Additionally, P53 HCN significantly decreased the relative risk with respect to both OS (HR = 0.5; *p* = 0.019) and PFS (HR = 0.5; *p* = 0.02).

### 3.6. Summary of Results

The group with only radiotherapy: Strong negative prognostic factors of OS such as 1q HCN, BCR HCN, and RB1 HCN were confirmed in this group and, furthermore 1q HCN and 22q12.2 HCN was found to be a significant negative prognostic factor of PFS.

The group with chemoradiotherapy: Negative clinical factors in the chemoradiotherapy group were male gender, high Karnofsky score, older age, higher expression of Ki67, and 22q12.2 HCN status. On the other hand, genetic markers such as CCND1 HCN, 19q13 HCN, MDM2 HCN, and p53 HCN indicate a positive influence on both OS and PFS in the chemoradiotherapy group. 

The group with no therapy: Although the group of patients comprised only a small number of participants, male gender, smoking status, and CCND1 HCN were marked as negative prognostic factors in this group.

All patient cohort: Male gender, RB1 HCN, and 22q12.2 were confirmed to be negative prognostic markers in the group of all patients. On the contrary, EGFR1 HCN, p53 HCN, CCND1 HCN, and 19q13 HCN were positive prognostic factors.

## 4. Discussion

Despite considerable efforts to clarify the molecular basis of GBM, the functional roles of known key genes and molecular markers are still unclear [16,17,18]. Previous studies have sought to identify distinct molecular signatures of diffuse gliomas and thereby reveal clinically relevant or functionally distinct GBM subclasses, but clinical applications for such signatures remain largely elusive [19,20,21,22,23]. Another problem is that genetic abnormalities have been defined rather ambiguously in many previous studies. Furthermore, most previous studies included patients who had undergone biopsy as well as resection [6,24,25,26,27], whereas our study examined only patients who underwent resection with the defined radicality. This should reduce heterogeneity and help reveal the effects of specific genetic factors. Analysis of patient records indicated that the main reason for omitting oncotherapy was poor neurological status (KS < 60) after surgery. Indications for sole radiotherapy and interruption of preliminary chemoradiotherapy were poor neurological status (KS ≈ 60) after surgery and rapid clinical deterioration during oncotherapy. Because the group of patients not receiving oncological treatment was very small (*n* = 24), the multivariate analysis for this group did not converge adequately and it was not possible to obtain HR values for OS or PFS.

The best outcomes for glioma patients are achieved through aggressive multimodal therapy involving safe and maximal resection followed by chemoradiotherapy [28]. In the studied cohort, every eligible patient who had undergone defined resection was reevaluated for oncotherapy suitability.

Each of the groups was distinguished by the survival and an occurrence of the prognostic factors. GBM wt patients receiving no oncological treatment or only radiotherapy are characterized by poor prognosis with substantial tumor growth [29]. Accordingly, our statistical analysis revealed very few positive prognostic factors in the group without oncotherapy. We, therefore, conclude that the course of the disease is effectively predetermined. This highlights the urgent need for new treatment strategies.

The main goal of most reported GBM studies has been to identify clinically relevant biomarkers with potential applications in medical practice, such as IDH [11]. In this work, we focused on selected genetic markers in an IDH wts GBM patient cohort and investigated their influence on disease evolution and its dependence on therapeutic modality. 

CCND1 HCN emerged as a clear positive marker of prolonged OS and PFS in the chemoradiotherapy group, although the opposite was observed in patients receiving neither chemotherapy nor radiotherapy. This could be due to increased chemosensitivity in tumors with elevated expression of this gene [30]. The CCND1 gene regulates the G1-S cell cycle phase transition and is thus involved in regulating cell proliferation and differentiation [31]. 

Increased MDM2 expression is known to block p53 activity, leading to uncontrolled glial cell proliferation and brain oncogenesis31. Surprisingly, our results indicated that MDM2 amplification had positive effects on survival in both the chemoradiotherapy group and the full patient cohort. Additionally, p53 amplification had positive effects on survival in all studied patient groups. This strongly suggests that the p53-MDM2 regulatory loop is involved in gliomagenesis which is known to be a complex process. 

Our results also revealed a negative effect of 22q12.2 polysomy on PFS across groups, which may warrant further investigation. Negative effects of loss of 22q on glioma progression have been reported previously [32,33,34], and 22q12.2 polysomy was identified as a malignant component in a study on ganglioglioma. Interestingly, this chromosomal region contains the EWSR1 gene, which causes Ewing sarcoma as well as neuroectodermal and other tumors [35,36].

The 1p/19q codeletion is a signature of oligodendroglioma, which has a relatively good prognosis. Additionally, GBM is often characterized by co-gain of chromosomes 1/19 and 19/20, both of which have been linked to superior outcomes [37,38]. However, this effect was not observed in our cohort, possibly because of the opposing effects of other genetic aberrations with negative effects on prognosis, such as EGFR1 amplification [39]. 

Pyrosequencing studies have shown that extensive MGMT methylation is associated with longer OS and PFS in GBM IDH wt patients, suggesting a possible beneficial effect of DNA alkylating chemotherapeutic strategies [40,41]. Marchi et al. showed that MGMT methylation extended OS and PFS in patients who had undergone gross total resection followed by adjuvant chemoradiotherapy. However, no such effect was observed when comparing our cohort of resected GBM patients receiving chemoradiotherapy to the other patient groups examined herein, even though the same molecular analysis protocol was used in both cases [42]. MGMT methylation status did not affect the clinical treatment given to the GBM patients; the Stupp protocol was considered the gold standard regardless of MGMT methylation. 

To our knowledge, no previous publications on GBM included cut-off values for the markers considered here. Therefore, an important objective of this work was to establish LCN and HCN values for each marker that could be used in subsequent studies in this area. 

Our results confirmed the prognostic value of clinical factors such as age, KS, smoking, and the applied therapeutic modalities. The effect of smoking may be partly due to nicotine-induced stimulation of malignancy in glioma cells, as suggested by a recent experimental study. However, other studies on this topic have yielded more ambiguous results [4,5,43]. Our results indicate that smoking is associated with shorter OS and PFS in all patient groups. The smokers in the patient cohort were predominantly male, which may explain the observed effect of gender on OS and PFS: males exhibited shorter survival than females. This effect warrants further investigation.

This study focused on the dominant IDH wt group of GBM patients, who have a very unfavorable prognosis. We confirmed the heterogeneity of IDH wt glioblastomas and investigated how differences in their molecular backgrounds influence survival. Although a standard chemotherapy regime (the Stupp protocol) is currently recommended for all GBM IDH wt patients, the optimal therapeutic strategy remains unknown. Understanding how various biomarkers predict treatment responses and outcomes will be vital for developing effective personalized treatment strategies for GBM patients [17,22,23,44]. 

## 5. Conclusions

Many studies have investigated prognostic factors for GBM patients with IDH mutations, for which prognosis is comparatively favorable. But the more frequent and deadlier IDH wt tumors have received less attention. We studied a cohort of IDH wt glioblastoma patients whose IDH status was determined in accordance with the WHO recommendations of 2016 [11,12]. The initial treatment strategy for this cohort was maximal radical and safe tumor resection, if possible, followed by oncotherapy. IDH wt gliomas have traditionally been regarded as a homogenous and unfavorable histological subtype with limited response to oncotherapy, making nihilistic management preferable. However, clinical outcomes for this diffuse glioma type are actually characterized by vast heterogeneity. The GBM wt subgroups with the poorest prognosis were characterized by an absence of positive prognostic factors and rapid tumor growth. Patients in these subgroups were those who received no oncotherapy or only radiotherapy. A crucial question of this study was: “How did pre-existing factors in resected GBM wt modify patient eligibility for a followed oncotherapy” Our aim was to identify clinical and biologically relevant prognostic factors for IDH wt GBM, to propose biological mechanisms explaining why these factors affect prognosis, and to identify clinically relevant IDH wt GBM subgroups. Knowledge of such factors and their impact on prognosis could facilitate decision-making about how aggressive the oncological strategy for a given patient should be, and whether reoperation is warranted, among other things. As such, the results presented here represent a first step towards the personalization of treatment for IDH wt GBM patients because they relate specific molecular markers to survival in this patient cohort. 

## Figures and Tables

**Table 1:** Primers and probes used to detect MGMT, E-cadherin, and Alu-M5 promoter methylation.

Gene	Primer	DNA Sequence	Final Concentration (µM)
MGMT promoter methylation	Forward	5′-CGAATATACTAAAACAACCCGCG-3′	1.0
	Reverse	5′-GTATTTTTTCGGGAGCGAGGC-3′	1.0
	Probe	FAM-BHQ-CAAATCCTCGCGATACGCACCGTTTACG	0.2
E-cadherin promoter methylation	Forward	5′-AATTTTAGGTTAGAGGGTTATCGCGT-3′	1.0
	Reverse	5′-TCCCCAAAACGAAACTAACGAC-3′	1.0
	Probe	FAM-BHQ-CGCCCACCCGACCTCGCAT	0.2
Alu-M5 promoter methylation	Forward	5′-GGTATGATGGCGTATGTTTGT-3′	0.17
	Reverse	5′-GACTCACCACAACTTCCAC-3′	0.17
	Probe	FAM-BHQ-AAACGATTCTCCTACCTCAACCTCCCGAA	0.03

**Table 2:** Demographic data for the patient cohort and information on the prevalence of low and high copy numbers of the studied markers.

Characteristics	Level	No Therapy	RT	CHT+RT	All Patients
Gender	F	10/24 (41.7%)	25/50 (50%)	17/58 (29.3%)	52/132 (39.4%)
	M	14/24 (58.3%)	25/50 (50%)	41/58 (70.7%)	80/132 (60.6%)
GBM location	F	8/24 (33.3%)	14/50 (28%)	20/58 (34.5%)	42/132 (31.8%)
	O	3/24 (12.5%)	10/50 (20%)	10/58 (17.2%)	23/132 (17.4%)
	P	2/24 (8.3%)	9/50 (18%)	13/58 (22.4%)	24/132 (18.2%)
	T	10/24 (41.7%)	15/50 (30%)	12/58 (20.7%)	37/132 (28%)
	other	1/24 (4.2%)	2/50 (4%)	3/58 (5.2%)	6/132 (4.5%)
Smoker *	No	9/24 (37.5%)	33/50 (66%)	41/58 (70.7%)	83/132 (62.9%)
	Yes	15/24 (62.5%)	17/50 (34%)	17/58 (29.3%)	49/132 (37.1%)
MGMT	Not methylated	16/23 (69.6%)	31/48 (64.6%)	33/52 (63.5%)	80/123 (65%)
	Methylated	7/23 (30.4%)	17/48 (35.4%)	19/52 (36.5%)	43/123 (35%)
Age category *	≤55	2/24 (8.3%)	2/50 (4%)	19/58 (32.8%)	23/132 (17.4%)
	>55	22/24 (91.7%)	48/50 (96%)	39/58 (67.2%)	109/132 (82.6%)
Karnofsky score *	0–79	16/24 (66.7%)	18/50 (36%)	12/58 (20.7%)	46/132 (34.8%)
	80–100	8/24 (33.3%)	32/50 (64%)	46/58 (79.3%)	86/132 (65.2%)
Ki67	≤60	20/24 (83.3%)	41/49 (83.7%)	49/58 (84.5%)	110/131 (84%)
	>60	4/24 (16.7%)	8/49 (16.3%)	9/58 (15.5%)	21/131 (16%)
1q CN	≤2.02	5/15 (33.3%)	12/33 (36.4%)	13/39 (33.3%)	30/87 (34.5%)
	>2.02	10/15 (66.7%)	21/33 (63.6%)	26/39 (66.7%)	57/87 (65.5%)
22q CN	≤2.48	13/16 (81.2%)	30/39 (76.9%)	33/41 (80.5%)	76/96 (79.2%)
	>2.48	3/16 (18.8%)	9/39 (23.1%)	8/41 (19.5%)	20/96 (20.8%)
CEP7 CN	≤2.04	4/16 (25%)	4/41 (9.8%)	10/50 (20%)	18/107 (16.8%)
	>2.04	12/16 (75%)	37/41 (90.2%)	40/50 (80%)	89/107 (83.2%)
BCR CN	≤2.04	6/15 (40%)	11/31 (35.5%)	14/39 (35.9%)	31/85 (36.5%)
	>2.04	9/15 (60%)	20/31 (64.5%)	25/39 (64.1%)	54/85 (63.5%)
EGFR1 CN	≤3.39	6/16 (37.5%)	25/44 (56.8%)	26/53 (49.1%)	57/113 (50.4%)
	>3.39	10/16 (62.5%)	19/44 (43.2%)	27/53 (50.9%)	56/113 (49.6%)
9p21.3 CN	≤1.86	6/17 (35.3%)	10/41 (24.4%)	16/49 (32.7%)	32/107 (29.9%)
	>1.86	11/17 (64.7%)	31/41 (75.6%)	33/49 (67.3%)	75/107 (70.1%)
1p36.3 CN	≤2.13	12/20 (60%)	29/45 (64.4%)	43/52 (82.7%)	84/117 (71.8%)
	>2.13	8/20 (40%)	16/45 (35.6%)	9/52 (17.3%)	33/117 (28.2%)
13q12.11 CN	≤2.01	3/10 (30%)	6/31 (19.4%)	14/41 (34.1%)	23/82 (28%)
	>2.01	7/10 (70%)	25/31 (80.6%)	27/41 (65.9%)	59/82 (72%)
RB1 CN	≤2.26	14/16 (87.5%)	37/42 (88.1%)	42/49 (85.7%)	93/107 (86.9%)
	>2.26	2/16 (12.5%)	5/42 (11.9%)	7/49 (14.3%)	14/107 (13.1%)
P53 CN	≤2.42	14/18 (77.8%)	38/44 (86.4%)	41/52 (78.8%)	93/114 (81.6%)
	>2.42	4/18 (22.2%)	6/44 (13.6%)	11/52 (21.2%)	21/114 (18.4%)
10p11.1	≤2.11	13/14 (92.9%)	33/36 (91.7%)	35/47 (74.5%)	81/97 (83.5%)
	>2.11	1/14 (7.1%)	3/36 (8.3%)	12/47 (25.5%)	16/97 (16.5%)
19q13 CN	≤1.83	2/18 (11.1%)	4/43 (9.3%)	4/53 (7.5%)	10/114 (8.8%)
	>1.83	16/18 (88.9%)	39/43 (90.7%)	49/53 (92.5%)	104/114 (91.2%)
MDM2 CN	≤1.97	1/17 (5.9%)	5/42 (11.9%)	3/51 (5.9%)	9/110 (8.2%)
	>1.97	16/17 (94.1%)	37/42 (88.1%)	48/51 (94.1%)	101/110 (91.8%)
CCND1 CN	≤2.48	12/14 (85.7%)	29/32 (90.6%)	40/47 (85.1%)	81/93 (87.1%)
	>2.48	2/14 (14.3%)	3/32 (9.4%)	7/47 (14.9%)	12/93 (12.9%)

* test of independence (chi-squared or Fisher exact test), *p*-value < 0.05; RT—radiotherapy; CHT+RT—chemoradiotherapy; F—female; M—male; F—frontal; O—occipital; P—parietal; T—temporal; BCR—breakpoint cluster region; EGFR1—epidermal growth factor receptor 1; RB1—retinoblastoma gene 1; TP53—tumor protein P53; MDM2—mouse double minute 2 homolog; CCND1—cyclin D1; MGMT—O6-methylguanine-DNA methyltransferase.

## Data Availability

Due to privacy and confidentially patient data is not available. Part of the data was presented at the European Association of Neuro-Oncology (EANO) Annual virtual meeting in October, 2016.

## Supplementary Materials

The following are available online at https://www.mdpi.com/1718-7729/28/2/122/s1, Table S1: The influence of various parameters on OS and PFS in the full patient cohort and subgroups receiving specific treatment modalities based on a univariate Cox proportional hazard regression model and Kaplan-Meier estimates. Table S2: Hazard ratios measuring the influence of the analyzed demographic variables and markers on OS and PFS based on multivariate Cox proportional regression models with adjustments for the prognostic factors of age at diagnosis (≤55 as reference category vs. >55 years) and KS (≤80 as reference category vs. >80). Table S3: Results of fitted Cox proportional hazard models of OS, resp. PFS for selected clinical factors and molecular markers stratified by therapy and adjusted for categorized Karnofsky score and age. For each model and non-reference category, the hazard ratio estimates (point estimate as well as 95%-confidence interval) and *p*-value are presented. Column N represents the number of patients in each factor level subgroup. Results for adjusting factors are not presented. Significant results are red-colored.
